# Intercropping Pinto Peanut in Litchi Orchard Effectively Improved Soil Available Potassium Content, Optimized Soil Bacterial Community Structure, and Advanced Bacterial Community Diversity

**DOI:** 10.3389/fmicb.2022.868312

**Published:** 2022-05-12

**Authors:** Ya Zhao, Caibin Yan, Fuchu Hu, Zhiwen Luo, Shiqing Zhang, Min Xiao, Zhe Chen, Hongyan Fan

**Affiliations:** Key Laboratory of Tropical Fruit Tree Biology of Hainan Province, Ministry of Agriculture and Rural Affairs/Haikou Tropical Fruit Tree Scientific Observation and Experimental Station, Institute of Tropical Fruit Trees, Hainan Academy of Agricultural Sciences, Haikou, China

**Keywords:** intercropping Pinto peanut in litchi, soil properties, soil enzyme activity, soil bacterial community structure, soil bacterial diversity

## Abstract

Intercropping is widely used in agricultural production due to its capability of raising land productivity and providing an opportunity to achieve sustainable intensification of agriculture. In this study, soil samples from 10 to 20 cm depth of intercropping Pinto peanut in litchi orchard and litchi monoculture mode were established to determine soil attributes, enzyme activities, as well as the effect on soil bacterial diversity. On this basis, 16S rRNA V4-V5 region of soil bacterial communities in litchi/Pinto peanut intercropping (LP) mode and litchi monoculture mode (CK) was detected by the Illumina MiSeq sequencing platform. The results showed that the content of available potassium (AK) in LP was significantly higher than that in CK by 138.9%, and the content of available nitrogen (AN) in LP was significantly lower than that in CK by 19.6%. The soil enzyme activities were higher in LP as a whole, especially sucrase (SC) and acid protease (PT) were significantly higher by 154.4 and 76.5%, respectively. The absolute abundance and alpha diversity of soil microbiota were significantly higher in the intercropping group. Most importantly, endemic species with a significant difference in LP was higher by ~60 times compared to CK treatment. In the aspect of soil bacterial community structure, the dominant phyla of the two groups were *Acidobacteria, Proteobacteria, Chloroflexi*, and *Actinobacteria*. At the genus level, the absolute abundance of *Flavobacterium* and *Nitrososphaera* was significantly higher by 79.20 and 72.93%, respectively, while that of *Candidatus_Koribacter* was significantly lower with an amplitude of 62.24% in LP than in CK. Furthermore, the redundancy analysis (RDA) suggested that AK, which was highly associated with the dominant genera and phyla, is the vitally dominating environmental factors in LP groups, while in CK groups, it is AN and pH. In addition, PICRUSt2 analysis indicated that intercropping improved the metabolic activity of bacteria which can be correlated to the resistance of litchi root systems to soil-borne diseases. Overall, this study is expected to provide a theoretical basis and technical support for the healthy intercropping cultivation of litchi.

## Introduction

Litchi (*Litchi chinensis* Sonn.) is a kind of south subtropical fruit, which is rich in nutrition, and native to China (Hu et al., 2021; Jiang et al., 2021). *L. chinensis* Sonn. cv. “Feizixiao,” an early maturing litchi variety, is widely planted in Hainan Province (Feng et al., 2015). The flower quantity of this variety is large at the flowering stage, leading to preserve the fruit hardly as it consumes too much soil nutrients, and the normal growth of flowers and leaves is seriously affected by the lack of calcium and water in the soil (Lu et al., 2017). *Arachis pintoi*, also known as the Pinto peanut, is a tropical and subtropical leguminous perennial creeping herbaceous plant (Zhang et al., 2021). Some studies have shown that it is an excellent intergrowth plant in ecological orchard that can improve not only microclimate and control weed growth but also fruit quality (Vu et al., 2019; Phan et al., 2021).

Intercropping is an agroecological practice of simultaneously growing two or more crops near the same field (Bedoussac et al., 2014), which is widely used in crop production. A meta-analysis shows that exploiting species complementarities by intercropping maize and soybean enables major increases in land productivity with less N fertilizer use (Xu Z. et al., 2020), and the increased N use efficiency in intercropping can reduce the requirements for fossil-based N fertilizer by about 26% on a global scale (Jensen et al., 2020). Recently, intercropping has received an increasing attention due to its potential advantages in increasing yield stability and yield per unit area, reducing pest problems and requirements for agrochemicals, while stimulating biodiversity (Ma et al., 2017; Maitra et al., 2021). Rodriguez et al. (2020) reported that intercropping could increase the use of N-sources and reduce the external inputs of N fertilizers for simultaneous production of both cereals and grain legumes with cropping systems' diversification. Studies have shown that the absolute yield gains were the greatest for mixtures of maize with short-grain cereals or legumes compared with monocultures (Li et al., 2020a). Tang et al. (2021) suggested that sugarcane/peanut intercropping significantly boosted the content of total nitrogen (TN), available phosphorus (AP), and total potassium (TK) and enhanced the activity of acid phosphatase compared to monoculture. Moreover, soil properties, nutrient use efficiency, and soil microbial diversity were significantly improved in the intercropping system (Zhao et al., 2022).

Soil nutrient and soil enzyme activities are critical factors in ecosystem productivity (Brooker et al., 2015). Soil enzymes, as an indicator of soil quality, are essential to organic matter synthesis and degradation in soils (Burns et al., 2013), and play critical roles in catalyzing biochemical reactions during the decomposition of microorganisms and plants, where their debris and subsequent release of nutrients into the soil is made available to the plants (Ma et al., 2017). Intercropping is generally deemed, could improve species diversity, and might have increased overall ecosystem productivity and nutrient retention (Cong et al., 2015), as it is reported that activities of enzymes associated with decomposition were highly increased in intercropping pattern soil (Hauggaar-Nielsen and Jensen, 2005). Soil microorganisms are vital in the processes of soil nutrient cycling (Gao et al., 2021) and nutrient transformation (Gul et al., 2015), which is also highly correlated with soil enzyme (Acosta Martínez et al., 2003). In addition, plant growth and soil elemental C:N:P ratios are linked to soil microbial diversity (Yang et al., 2021). Although many studies of intercropping have been conducted which put emphasis on crop yield and its effect on soil properties, few studies have clearly demonstrated the interactions between the microbial community structure and the soil environment; especially, the effects of intercropping Pinto peanut in litchi orchard on soil nutrient, enzyme activities, and soil bacterial community structure and diversity remain unclear yet. In this paper, soil samples of litchi intercropped with Pinto peanut and of litchi monoculture from 10 to 20 cm soil layers were collected to elucidate the effect of intercropping on soil properties, enzyme activities, and soil bacterial community structure and diversity, using high-throughput sequencing on the Illumina MiSeq platform. In addition, the correlation between soil properties and soil microbial community was further analyzed to provide scientific theoretical guidance for compound cultivation mode.

## Materials and Methods

### Plant Materials

*Litchi* cv. “Feizixiao,” planted in 1998 with plant spacing of 4 × 5 m, was used as plant materials. The intercrop in this study, *A. pintoi* cv. “Reyan No. 12,” is one of the varieties of *A. pintoi*, which was introduced by the Center of International Agriculture Tropical (CIAT) in 1991 by the Chinese Academy of Tropical Agricultural Sciences (CATA), and it has the characteristics of acid resistance, adaptability, shading tolerance, and nutritional value (Bai et al., 2019). Pinto peanut was planted at 1.5 m from the base of the litchi stem in 2015 with a dense of 36 Pinto peanut seedlings per square meter of land.

### Site Description and Experimental Design

The experimental site is located in the litchi demonstration orchard of Yongfa Fruit Tree Base, Chengmai, Hainan Province, China (109°45′~110°15′E, 19°23′~20°01′N). This region is a tropical monsoon climate with abundant rainfall and sunshine, with an annual average temperature of 23.7°C, an extremely high temperature of 38°C, an extremely low temperature of 7°C, and a frost-free period throughout the whole year. The test site is red loam soil, the annual sunshine time is 2,060.6 h, and the annual average rainfall is 1,756 mm. The experiment was designed for two treatments, namely, the intercropping of litchi and Pinto peanut (LP) and monoculture of litchi (CK) (Supplementary Figures 1–5). All treatments were fertilized as the below management: Fertilized once in florescence stage (in January to March) by ENTEC (Germany) compound fertilizer with DMPP(3,4-dimethylpyrazole phosphate): include 9.8% ${\text{NO}}_{3}^{-}$, 12.2% ${\text{NH}}_{4}^{+}$, 4.0% total S, 7.0% P_2_O_5_ and 11.0% K_2_O, the same as below), 1 kg per tree; the secondary fertilization was applied in fruit-set period (in March to April): compound fertilizer (ENTEC), 1 kg per tree; the last time of fertilization was applied in June after the fruit was harvested, which there was a heavy compound fertilizer application in this stage: compound fertilizer (ENTEC), 2.5 kg per tree; and organic fertilizer (chicken dung), 20 kg per tree; all plots underwent other identical standardized management practices. There was no fertilization on the day of sampling.

### Soil Sampling and Analyses

#### Soil Sampling

The soil samples were collected in the vegetative period of *A. pintoi* in December of 2019. Briefly, soil samples from 10 to 20 cm depths were randomly collected from the junction of litchi root system and Pinto peanut (about 1.5 m from the base of the litchi stem) on four plots (i.e., from the east, west, south, and north of litchi stem) of 4 m^2^ (2 × 2 m) in intercropping and monoculture areas using the five-point sampling method. For each sample plot, the samples were replicated three times, resulting in a total of 12 soil samples (three replications for 4 sample plots) in each treatment. Subsequently, each plot soil sample was fully mixed to filter out impurities, such as plant roots and stones, using 1 mm mesh, and then finely grounded and shifted to ensure a uniform sample. The soil samples from the east, west, south, and north of the same litchi stem were mixed to a composite soil sample, and each treatment was replicated three times and grouped into LP and CK, respectively. The above samples were divided into three parts, namely, one was stored at −80°C for 16S rRNA gene analysis, one was air-dried for determining the soil chemical properties and soil enzyme activity, and the rest was stored at 4°C.

#### Soil Properties Analyses

Total soil organic carbon (SOC) was determined by the dichromate oxidation method (Gao et al., 2021; Wang X. et al., 2021), and TN was analyzed by the semi-micro Kjeldahl digestion method (Kachurina et al., 2000). The total phosphorus (TP) of the soil was extracted using H_2_SO_4_-HClO_4_ and then measured by the molybdenum blue method (710 nm) using an ultraviolet spectrophotometer (Hitachi UV2300) (Olsen and Sommers, 1982); TK and available nitrogen (AN) of soil were measured by the alkali fusion-flame photometer method (Xi et al., 2019); soil nitrate nitrogen (NO3^−^-N) and ammonium nitrogen (NH4^+^-N) concentrations were measured in a 1-M potassium chloride (KCl) solution [soil: solution = 1:10 (w/v)] using a segmented-flow analyzer after extraction (Wang X. et al., 2021). Soil AP and available potassium (AK) were measured *via* the NaHCO_3_-molybdenum antimony colorimetric method and NH_4_OAc-flame photometer method (Bao, 199), respectively. The soil moisture was measured by the traditional 105° drying weighing method (Xu D. et al., 2020). Soil pH was determined at a soil-to-water ratio of 1:2.5 using an S200 K pH meter (Mettler-Toledo International Inc., Shanghai, China).

#### Determination of Soil Enzyme Activities

The activities of soil sucrase (s-SC) were determined by the 3,5-dinitrosalicylic acid (DNSA) method (Sun et al., 2020), soil urease (s-UE) was determined by the sodium hypochlorite colorimetric method (Chen and Huang, 2020), soil catalase (s-CAT) was determined by the photometer colorimetric method (Alef and Nannipieri, 1995), soil polyphenol oxidase (s-PPO) was detected with the autoxidation of pyrogallol method, and soil protease (s-PT) was determined by casein hydrolysis (Sun et al., 2020).

#### Soil DNA Extraction, Absolute Amplification, and Pyrosequencing of 16S rRNA

The total genomic DNA was extracted using the FastDNA^®^ SPIN Kit for Soil DNA Extraction (MP Biomedicals, Santa Ana, CA) according to the manufacturer's instructions. The DNA was purified through Agencourt AMPureXPPCR Purification Beads (Beckman Coulter, USA). The integrity of genomic DNA was detected through agarose gel electrophoresis, and the concentration and purity of genomic DNA were detected *via* Qubit 3.0 Spectrophotometer. The V3-V4 hypervariable regions of the 16S rRNA gene and spike-ins were amplified with the primers 341F (5′-CCTACGGGNGGCWGCAG-3′)/805R (5′-GACTACHVGGGTATCTAATCC-3′) (Kataoka et al., 2019). The PCR amplification reaction was performed in triplicate in a total volume of 10μl. The reaction mixture consisted of 1 μl of 10 × Toptaq Buffer, 0.2 μl of Toptaq DNA Polymerase, 0.2 μl of each primer (10 μM), and 3 μl of template DNA. The following thermal cycling conditions were used: initial denaturation at 94°C for 2 min, 25 cycles of denaturation at 95°C for 30 s, primer annealing at 55°C for 30 s, extension at 72°C for 1 min, and a final extension at 72°C for 10 min. The 16S rRNA gene amplicon was sequenced on the Illumina MiSeq platform at Genesky Biotechnologies, Inc. (Shanghai, China).

### Statistical Analysis

The raw sequencing data were processed and trimmed using Quantitative Insights Into Microbial Ecology (QIIME) and usearch software to remove the low-quality sequences (quality score < 20); primers, barcodes, adaptors (Caporaso et al., 2010), and chimeras were detected and removed using the UCHIME algorithm (Edgar et al., 2011). The remaining high-quality sequences were clustered into operational taxonomic units (OTUs), with a 97% similarity cutoff value. Alpha diversity, including the Chao1, ACE, Shannon, Simpson, and coverage indices, was calculated using the Mothur software and R software version 3.3.1 based on the obtained OTUs. The beta diversity analysis consisted of a principal coordinate analysis (PCoA) and non-metric multidimensional scaling (NMDS). The R software version 3.3.1 (vegan package) was used in PCoA and NMDS analysis based on the Bray–Curtis distance, Jaccard, and unweighted and weighted UniFrac metrics. Linear discriminant analysis (LDA) effect size (LEfSe) was performed to identify the biomarkers between groups where the threshold score of LDA was 2. The functions of species in the gut microbiota of both the groups were predicted and analyzed on the basis of amplified sequence data, using the PICRUSt2 analysis tool and Kyoto Encyclopedia of Genes and Genomes (KEGG) database (https://www.genome.jp/kegg/pathway.html).

Correlations between the soil properties and soil enzyme activities were determined using SPSS version 20.0 (SPSS Inc. Chicago, IL, USA). Canonical correspondence analysis (CCA) was used to analyze the relationships between the bacterial community and environmental factors by Canoco 5 in conjunction with the chi-square test. Statistical significance differences among the samples were calculated *via* two-way analysis of variance (ANOVA) in conjunction with a *t*-test, and a value of *P* < 0.05 was considered to be statistically significant.

## Results

### Effect of Intercropping on Soil Attributes

Soil attributes of the treatments are shown in Table 1. In general, there were no significant differences between LP and CK in terms of soil parameters, except for AN, which was higher by 19.6% in CK, and AK, which was significantly higher in LP by 138.9% (*p* ≤ 0.05) (Table 1).

**Table 1:** Soil properties of LP soils and CK soils.

**Group**	**SOC (g/kg)**	**TN (g/kg)**	**TP (g/kg)**	**TK (g/kg)**	**AP (mg/kg)**	**AN (mg/kg)**	**AK (mg/kg)**	**SA**	**pH**
CK	0.05 ± 0.00	1.19 ± 0.13	0.86 ± 0.01	2.21 ± 0.19	159.0 ± 5.93	59.50 ± 2.18^a^	247.4 ± 23.49^a^	0.97 ± 0.00	5.75 ± 0.06
LP	0.05 ± 0.00	1.26 ± 0.14	0.71 ± 0.04	2.28 ± 0.23	145.8 ± 5.23	47.83 ± 0.93^b^	591.0 ± 26.02^b^	0.98 ± 0.00	5.63 ± 0.08

*The different letter in the same column represents significant difference (Duncan test, p ≤ 0.05, n = 3) between groups. The number behind the plus-minus sign represents SE. CK, monoculture of litchi; LP, intercropping of Litchi and Pinto peanut; SOC, soil organic carbon; TN, total nitrogen; TP, total phosphorus; TK, total potassium; AN, available nitrogen; AP, available phosphorus; AK, available potassium; SA, soil moisture*.

### Effect of Intercropping on Soil Enzyme Activities

Compared to CK treatment, the soil enzyme activities were significantly higher in the intercropping group, especially in SC and acid PT, which was significantly higher by 154.4 and 76.5%, respectively (*p* ≤ 0.05). As shown in Table 2, the s-PPO, s-UE, and s-CAT in the intercropping group, compared to the CK group, were significantly higher by 32.6, 15.0, and 16.9%, respectively (*p* ≤ 0.05); meanwhile, the neutral PT and alkaline PT were not significantly different between LP and CK.

**Table 2:** Soil enzyme activities in the LP soils and CK soils.

**Group**	**Sucrase**	**Urease**	**Catalase**	**Polyphenol oxidase**	**Acid protease**	**Neutral protease**	**Alkaline protease**
	**(mg/d/g)**	**(μg/d /g)**	**(μmol/d/g)**	**(mg/d/g)**	**(mg/d/g)**	**(mg/d/g)**	**(mg/d/g)**
CK	4.19 ± 0.27^a^	899.7 ± 24.04^a^	52.66 ± 2.60^a^	49.59 ± 3.94^a^	0.98 ± 0.03^a^	3.35 ± 0.08	2.33 ± 0.08
LP	10.66 ± 0.91^b^	1034.7 ± 22.55^b^	61.57 ± 0.67^b^	65.75 ± 0.43^b^	1.73 ± 0.15^b^	3.41 ± 0.19	2.53 ± 0.22

*The different letter in the same column represents significant difference (Duncan test, p ≤ 0.05, n = 3) between groups. The number behind the plus-minus sign represents SE. CK, monoculture of litchi; LP, intercropping of Litchi and Pinto peanut*.

### Effects of Intercropping on Soil Bacterial Community

#### Effects of Intercropping on the Abundances of Soil Bacteria

**Figure 1:**
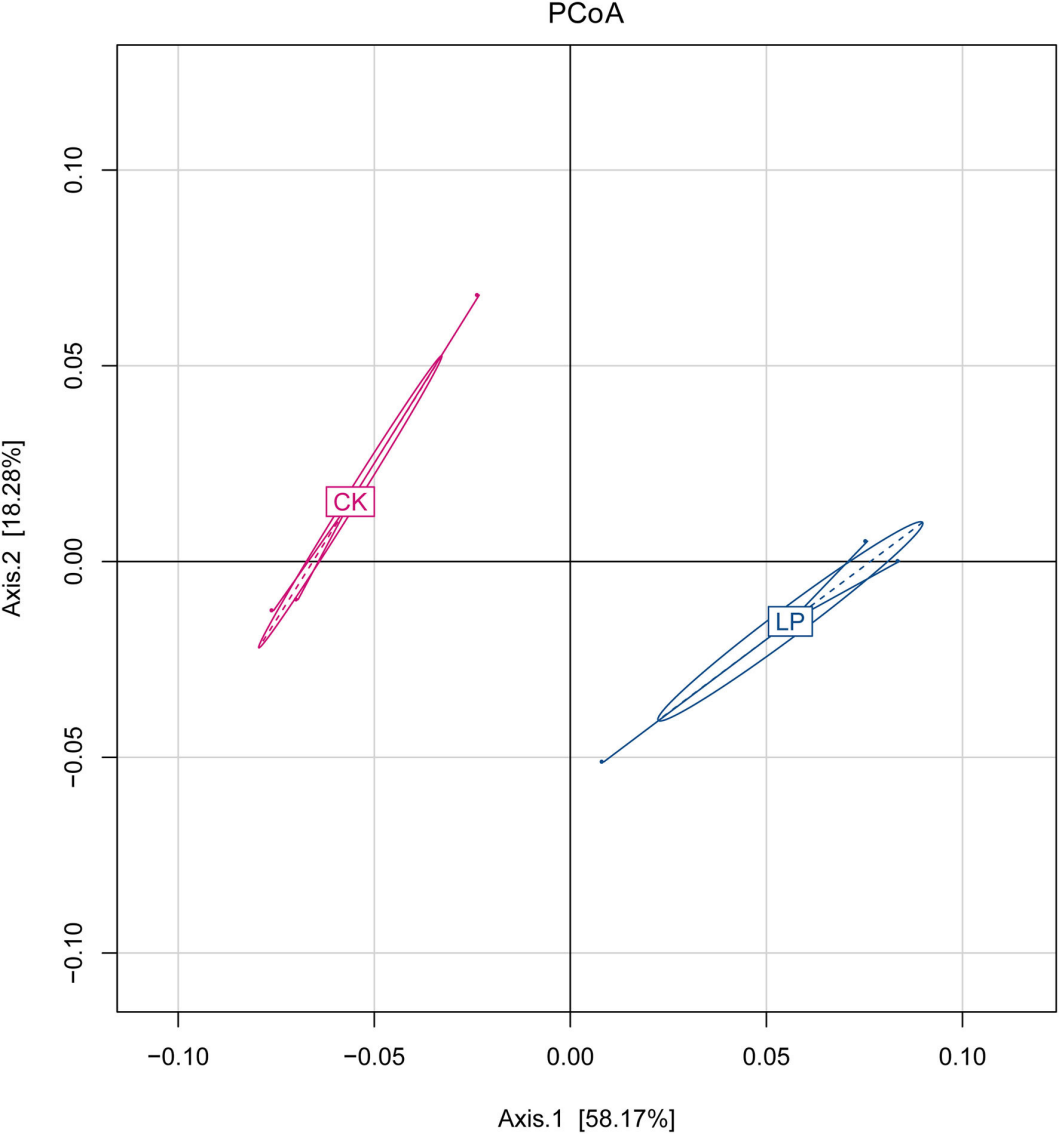
Principal coordinate analysis (PCoA). Axes 1 and 2 are two main components with the most interpretation of differences between samples. CK: monoculture of litchi; LP: intercropping of litchi and Pinto peanut.

**Figure 2:**
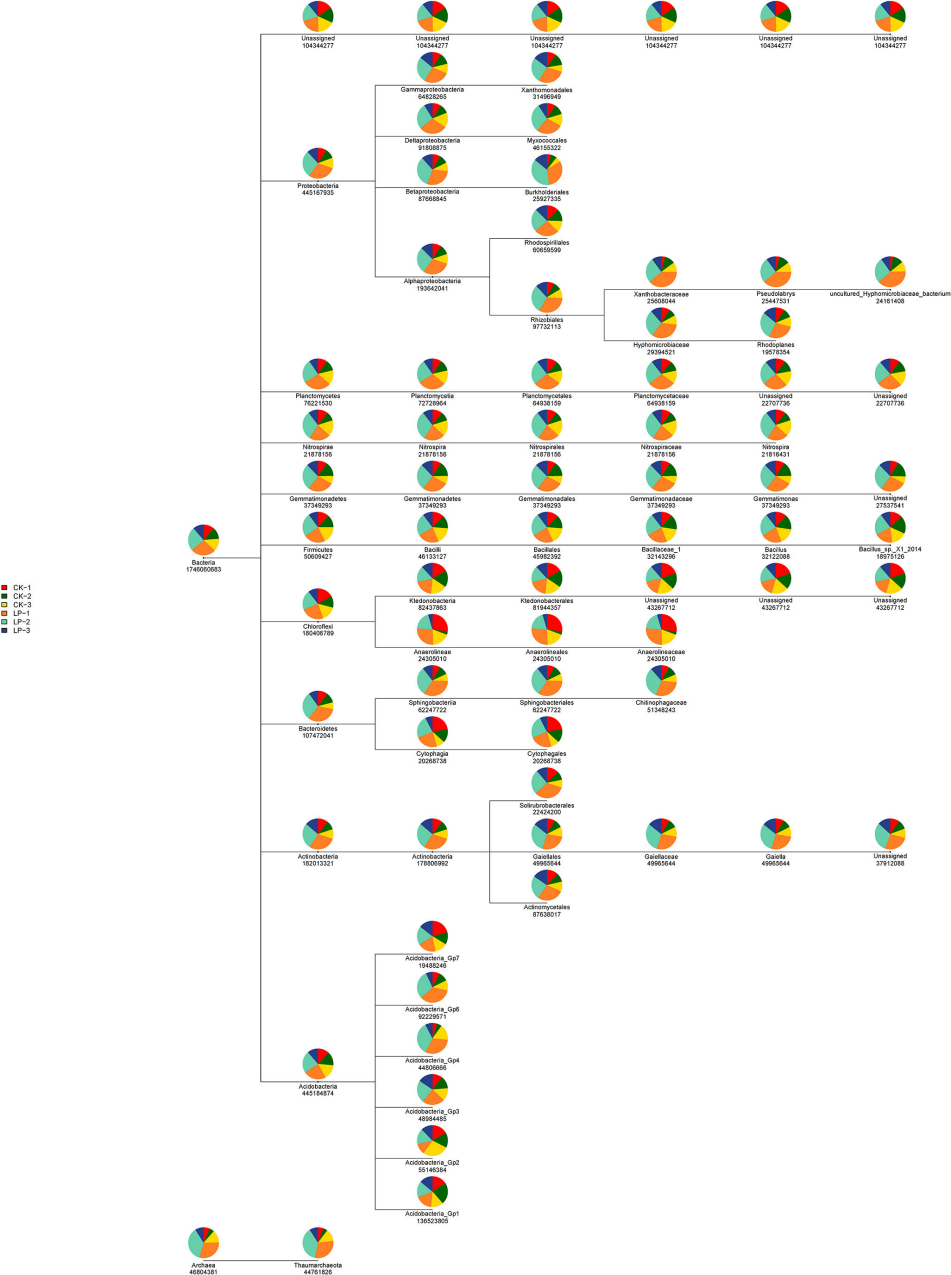
The phylogenetic tree of intercropping and monoculture groups. The phylogenetic tree, from the leftmost root node to the rightmost leaf node, each layer represents a classification level from boundary to species level. For species at each level, a pie chart is used to show the proportion of the species in each sample (the proportion > 1%), different colors represent different samples, and the larger the fan area of a color, the larger the number of sequences. The numbers under the circle represent the sum of the sequences of the species in all samples.

The results of PCoA are displayed in Figure 1, in which the bacterial communities of the monoculture soil (CK) were clearly separated from those of intercropping treatment, indicating that the two groups exhibited obviously distinct clustering of microbiota composition and the microbial structure showed a significant difference between the intercropping treatment and the monoculture treatment. In addition, the phylogenetic tree showed that the absolute abundance of bacterial community of three intercropping soil samples was significantly higher than that of monoculture at all classification levels (Figure 2), suggesting that the total species absolute abundance in soil was highly increased in the intercropping group. The above conclusion is also confirmed by the analysis results of OUT copies (Table 3).

**Table 3:** Richness and diversity indices of different treatment groups.

**Groups**	**Indexes**					
	**Observed/OTUs**	**Chao1**	**ACE**	**Shannon**	**Simpson**	**Coverage**
CK	5922.0 ± 528.80	6398.7 ± 499.29^a^	6367.4 ± 478.88^a^	6.82 ± 0.15	0.0032 ± 0.00^a^	0.998 ± 0.00
LP	6058.0 ± 312.65	6637.9 ± 189.63^b^	6590.6 ± 212.19^b^	6.90 ± 0.03	0.0030 ± 0.00^b^	0.996 ± 0.00

*The different letter in the same column represents significant difference (Duncan test, p ≤ 0.05, n = 3) between groups. The number behind the plus-minus sign represents SE. CK, monoculture of litchi; LP, intercropping of Litchi and Pinto peanut*.

#### Diversity Analysis of Soil Bacterial Community

Alpha diversity analysis of soil bacterial communities in the two planting patterns indicated that intercropping Pinto peanut in litchi orchard could significantly improve soil bacterial richness and diversity (Table 3). Compared with the control group, Chao1 and ACE indices, representing the community richness of the sample, were significantly higher in the intercropping group, with the amplitude of 3.7 and 3.5%, respectively. In addition, the Simpson index, which was negatively correlated with the diversity of community structure, was significantly lower by 6.6%. These results suggested that intercropping could promote the richness and diversity of soil community structure of microbiota.

**Figure 3:**
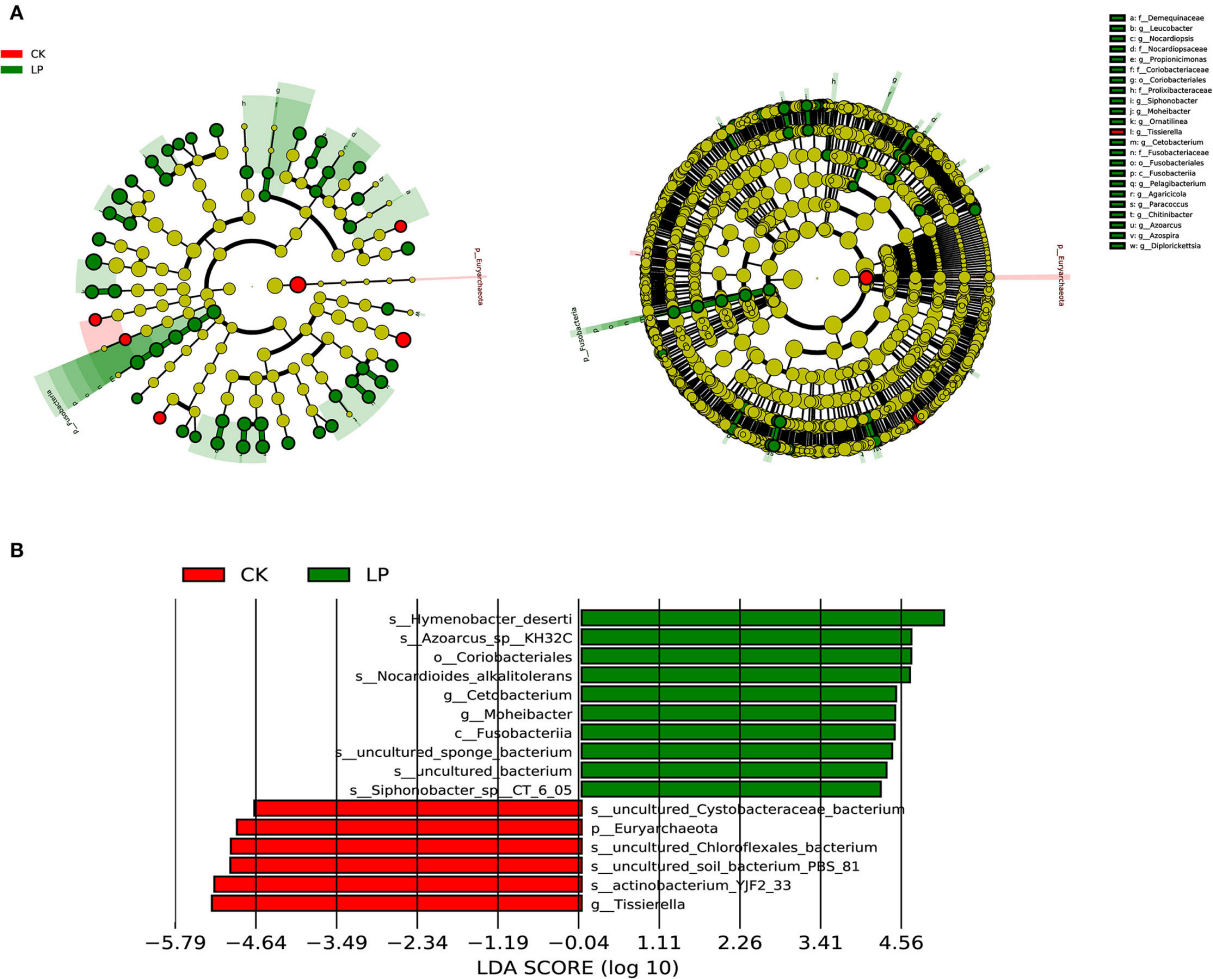
Taxa with different abundances according to linear discriminant analysis (LDA) effect size (LEfSe) analysis in the intercropping and CK groups. **(A)** Cladogram generated by LEfSe. Statistically significant differences (LDA scores >2) in absolute abundance of top 50 taxa with the minimum *P*-value between the intercropping and CK groups, *p* ≤ 0.05. Red and green nodes indicate enriched taxa in the intercropping and CK groups, respectively. The diameter of each node shows the absolute abundance of each taxon and is proportional to the observed effect size. The circle of evolutionary branching maps from internal to external radiation represents the classification level from the phyla to the species, and notes of species markers in each layer represent the classification level from the phyla to the species from the outside in. **(B)** Histogram of the LDA scores (>2) computed for the top 10 taxa with minimum *P*-value. Red and green bars indicate enriched taxa in the intercropping and CK groups, respectively.

The LEfSe analysis was performed to identify the specific taxa with consistently altered abundance in constipation. A cladogram for all the taxonomic levels' abundance is shown in Figure 3A, in which species with significantly higher abundance (endemic species with a significant difference) in the intercropping group were higher than that in CK treatment in all the taxonomic levels. A total of 353 taxa (from phylum to species) were identified with LDA scores of >2 and *P*-value of > 0.05 in the intercropping group, which increased ~60 times compared to CK treatment that only 6 taxa (from phylum to species) were identified. The top 10 taxa with the highest LDA scores in each group are shown in Figure 3B, indicating that the intercropping group was representatively enriched with species *Hymenobacter deserti, Azoarcus-sp-KH32C* and *Nocardioides alkalitolerans*, genus *Cetobacterium* and *Moheibacter*, order *Coriobacteriales*, and class *Fusobacteriia*, while the monoculture groups were enriched with species *Cystobacteraceae-bacterium, Chloroflexales_bacterium, soil-bacterium-PBS-81, actinobacterium-YJF2-33*, genus *Tissierella*, and phylum *Euryarchaeota*. The diversity of the above species composition with significantly high abundance might be the crucial player involved in causing community structure differences between groups. The above results indicated that intercropping litchi with peanut could greatly improve the diversity of bacterial community structure in the soil.

#### Bacterial Community Structure of Soil Under Intercropping Treatment

##### Compositional Analysis of Bacterial Community at the Phylum Level

**Figure 4:**
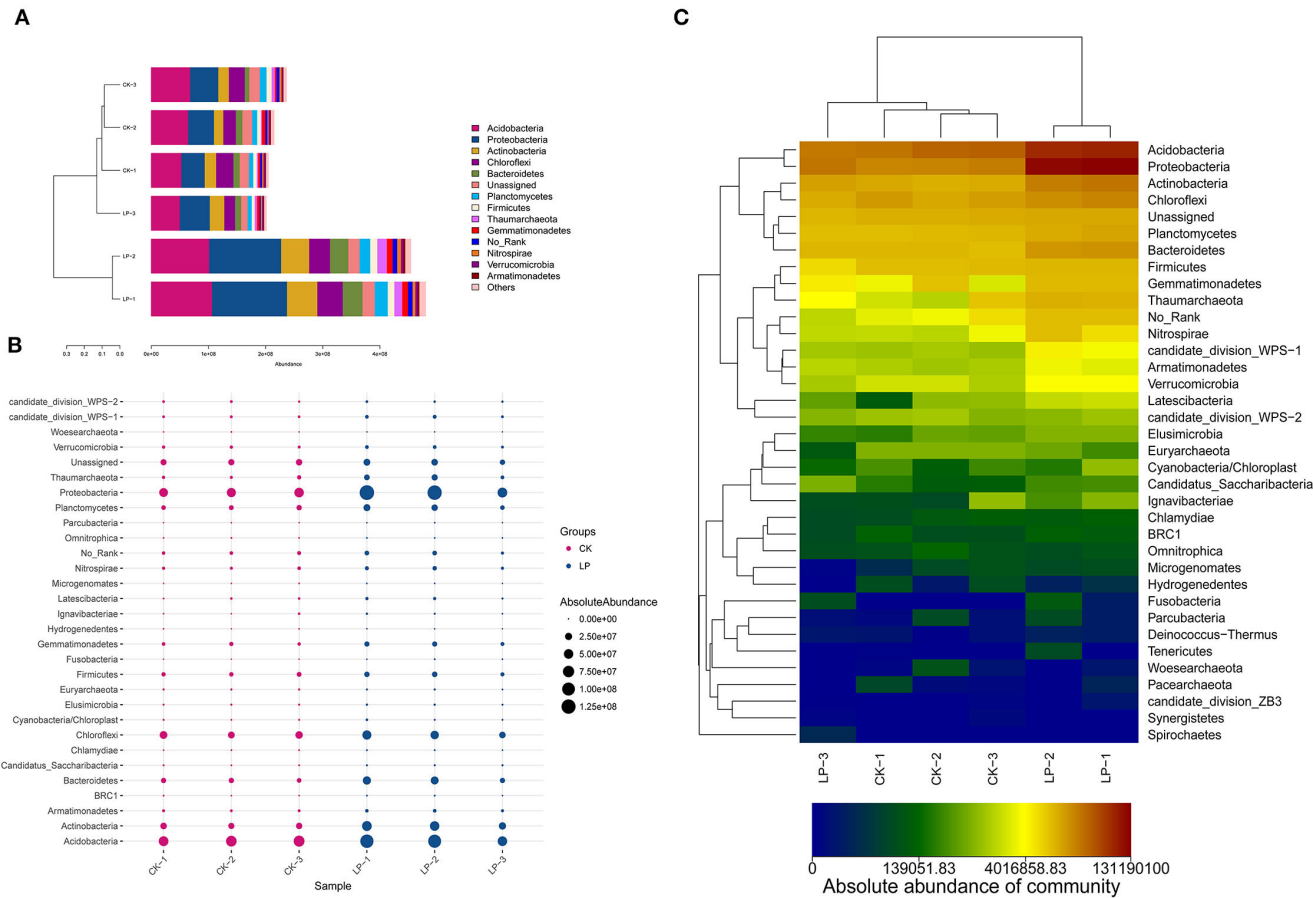
Compositional analysis of bacterial community at the phylum level. **(A)** Bar plot of the compositional alteration of the soil bacteria in the intercropping group (the proportion > 1%). **(B)** The bubble chart with changes of absolute abundance (>1%) in soil bacteria between LP and CK groups. **(C)** Heat map of soil different bacterial composition at the phylum level.

As shown in Figure 4, there are 12 phyla groups with the average absolute abundance of soil bacterial community >1% in both intercropping and CK groups. The dominant bacterial phyla across all soil samples were the same, which included *Acidobacteria, Proteobacteria, Chloroflexi*, and *Actinobacteria*, with absolute abundances ranging from 22.78 to 28.38%, 20.63 to 27.26%, 8.81 to 12.23%, and 8.33 to 11.21% (Figure 4A). There are 6 phyla, having significant differences in their absolute abundances, between two groups (Figure 4C); *Proteobacteria, Acidobacteria, Actinobacteria, Chloroflex, Bacteroidetes*, and *Planctomycetes* were significantly higher in the intercropping group when compared with CK treatment (Figure 4B).

##### Compositional Analysis of Bacterial Community at the Genus Level

**Figure 5:**
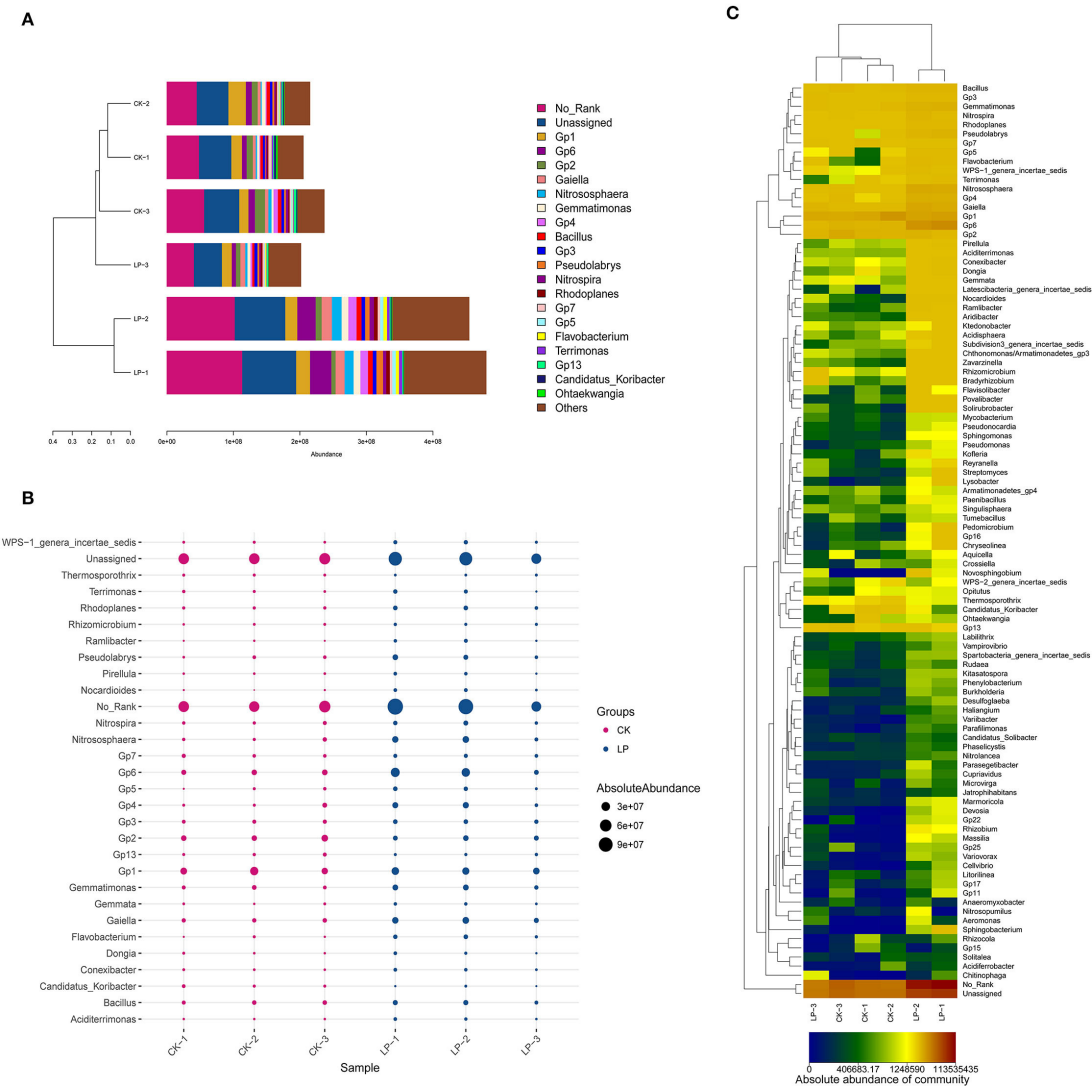
Compositional analysis of bacterial community at the genus level. **(A)** Bar plot of the compositional alteration of the soil bacteria in the intercropping group (the proportion > 1%). **(B)** The bubble chart with changes of absolute abundance (>1%) in soil bacteria between LP and CK groups. **(C)** Heat map of soil different bacterial compositions at the genus level.

There were 16 genera with an average absolute abundance >1% of the tested bacterial flora, including 12 in the CK group and 14 in the intercropping group (Figure 5A). The dominant bacterial genera across all soil samples were the same, which included *Acidobacteria*_*Gp1* (8.5–4.72%), _*Gp6* (3.98–5.82%), _*Gp2* (5.03–1.95%), _*Gp4* (1.44–2.33%), _*Gp3* (1.64–1.36%), *Gaiella* (2.12–3.17%), *Bacillus* (2.16–1.58%), *Nitrososphaera* (1.3–2.78%), *Nitrospira* (1.22–1.22%), and *Gemmatimonas* (1.87–2.2%). Furthermore, *Gp1* had the highest absolute abundance in the CK group, while in the intercropping group, it was *Gp6*. As shown in Figure 5C, a total of 98 bacterial genera for the intercropping group were higher in absolute abundance, among which *Flavobacterium, Nitrososphaera, Pseudolabrys, GP4, Gaiella*, and *Gp6* were significantly higher by 79.20, 72.93, 66.17, 64.25, and 61.35%, respectively; but *Candidatus_Koribacter, Gp2*, and *GP1* were significantly lower, with a difference of 62.24, 32.97, and 4.02% compared to the CK group (Figure 5B).

#### Correlations of Soil Properties With Soil Bacterial Communities and Enzyme Activities

**Figure 6:**
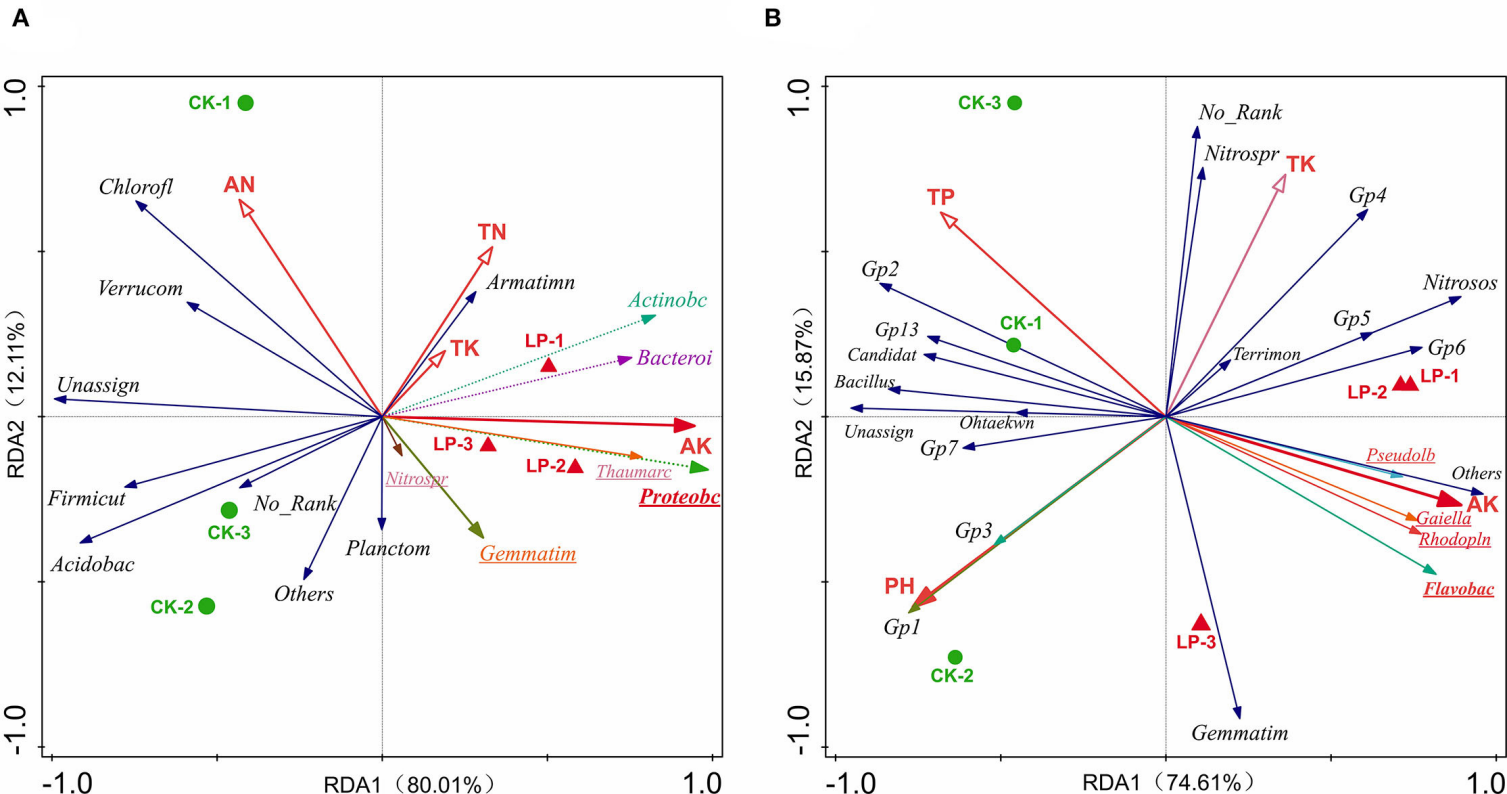
Redundancy analysis (RDA) for dominate soil bacterial communities from all samples associated with environmental variables at the phylum level **(A)** and genus level **(B)**, respectively. CK, monoculture of litchi; LP, intercropping of Litchi and Pinto peanut; TP, total phosphorus; TK, total potassium; AK, available potassium.

The effects of soil properties on the microbial communities were analyzed using redundancy analysis (RDA) (Figure 6). At the genus level, the first axes (RDA1) explained 74.61% of the variation, and the second axes (RDA2) explained 15.87% of the variation, which totally explained as high as 90.48% of the variation in bacterial communities. Similarly, the first axes (RDA1) explained 80.01% of the variation, and the second axes (RDA2) explained 12.11% of the variation, which totally explained as high as 92.12% of the variation in bacterial communities at the phylum level (Table 4). According to the conditional effects, the explanation of the variation of AK was the highest in both genus and phylum levels, with the amplitude of 61.0 and 71.7%, respectively. In summary, AK is the vitally dominating environmental factors, followed by pH, AP, and TK, in which these four indexes accounted for 96.80% of the total shift in microbial communities, which affected the soil microbial structure (Table 5). At the genus level, the dominating genera, namely, *Flavobac, Nitrosos, Rhodopln, Gaiella, Gp6, Pseudolb, Gp5*, and *Gp4*, were positively associated with AK but negatively correlated with pH. In addition, *Bacillus, Gp1, Gp2, Gp3*, and *Gp7* were positively associated with pH but negatively correlated with AK (Figure 6B). At the phylum level, the results of RDA analysis are consistent with those at the genus level (Figure 6A).

**Table 4:** Indexes of RDA analysis at phylum and genus levels.

**Statistic**	**Genus**				**Phylum**			
	**Axis 1**	**Axis 2**	**Axis 3**	**Axis 4**	**Axis 1**	**Axis 2**	**Axis 3**	**Axis 4**
Eigenvalues	0.7461	0.1587	0.0536	0.0037	0.8001	0.1211	0.0405	0.0056
Explained variation (cumulative)	74.61	90.48	95.83	96.20	80.01	92.12	96.17	96.73
Pseudo-canonical correlation	0.9999	1.0000	0.9861	0.9052	0.9984	0.9977	0.9975	0.6314
Explained fitted variation (cumulative)	77.56	94.05	99.62	100.00	82.71	95.23	99.42	100.00

**Table 5:** The RDA analysis of soil properties.

**Item**	**Genus**				**Phylum**			
		**AK**	**pH**	**TP**	**TK**	**AK**	**AN**	**TN**	**TK**
Conditional effects	Explains (%)	61.0	21.0	9.2	4.9	71.7	9.4	9.2	6.5
	pseudo-F	6.3	3.5	2.1	1.3	10.1	1.5	1.9	2.0
	P	0.056	0.042	0.176	0.368	0.066	0.258	0.24	0.346

*AK, available potassium; TP, total phosphorus; TK, total potassium; AN, available nitrogen; TN, total nitrogen*.

The soil samples of monoculture treatment (CK) were mainly distributed in the second and third quadrants, which indicated that the difference in bacterial community structure was mainly caused by the changes in pH and TP, while it is in the first and fourth quadrants of the soil sample distribution of intercropping group (LP) that the bacterial community structure was mainly affected by AK and TK (Figures 6A,B). All the above results were highly consistent with the previous studies on soil properties, which implied that bacterial community structures were significantly affected by intercropping treatment.

Furthermore, the Pearson correlation coefficients were also calculated between the soil properties and the soil enzyme activity in which AK was significantly positively correlated with PPO, and UE and alkaline PT were significantly positively correlated with SOC and TN, respectively. Furthermore, AN was significantly negatively correlated with SC, UE, and CAT. In addition, the rise of soil pH significantly decreases the activity of UE and alkaline PT (Table 6). To sum up, intercropping could affect the soil microbial environment to augment the soil enzyme activity, and may be capable of improving the AK contents of soil.

**Table 6:** Correlations of soil properties with soil enzyme activity.

**Item**	**Sucrase**	**Urease**	**Catalase**	**Polyphenol oxidase**	**Acid protease**	**Neutral protease**	**Alkaline protease**
SOC	0.600	0.714	0.371	0.600	0.257	0.314	0.886^*^
TN	0.771	0.714	0.600	0.486	0.371	−0.029	0.829^*^
AN	−0.943^**^	−0.886^*^	−0.886^*^	−0.771	−0.771	0.029	−0.543
TP	−0.600	−0.543	−0.486	−0.371	−0.314	0.200	−0.429
AP	−0.257	−0.086	−0.486	−0.143	−0.543	0.086	0.486
TK	0.371	0.543	0.143	0.486	0.086	0.486	0.771
AK	0.771	0.886^*^	0.714	0.943^**^	0.771	0.486	0.371
SA	0.543	0.429	0.600	0.371	0.543	−0.257	0.257
pH	−0.771	−0.829^*^	−0.543	−0.657	−0.371	−0.200	−0.943^**^

*and **represent the significant correlation at p < 0.05 and p < 0.01, respectively. SOC, soil organic carbon; TN, total nitrogen; TP, total phosphorus; TK, total potassium; AN, available nitrogen; AP, available phosphorus; AK, available potassium; SA, soil moisture.

#### Functional Predictions for Soil Microbiota

**Figure 7:**
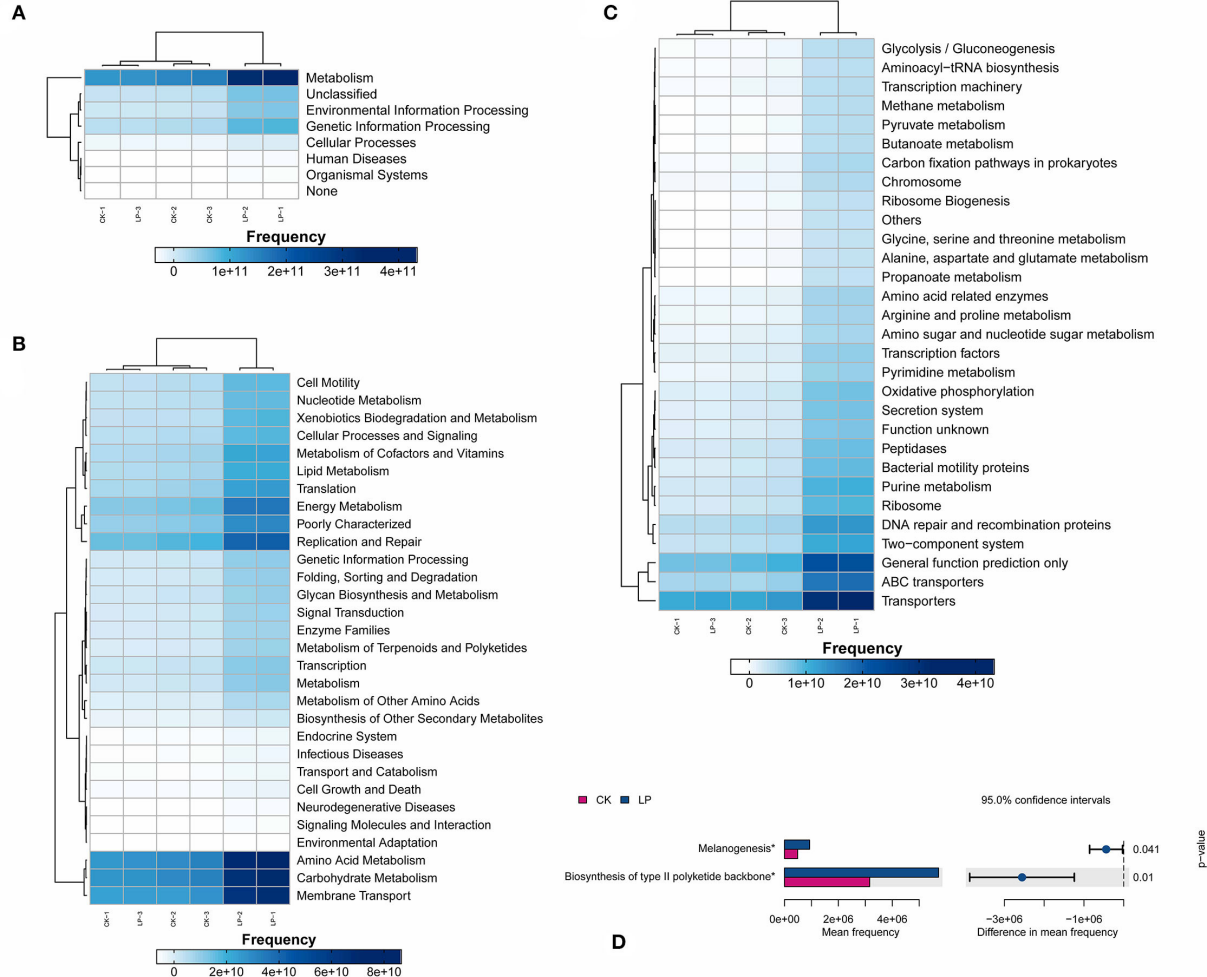
Functional predictions of the soil microbiota. **(A–C)** represent functional genes annotated in the KEGG database at levels 1–3, respectively; **(D)** The significantly different pathway genes between groups, *Significant correlation at *p* ≤ 0.05; CK, monoculture of litchi; LP, intercropping of litchi and Pinto peanut.

To identify the differences in the functional prediction between the two groups, the absolute abundances of functional genes in the two groups were compared using the PICRUSt2 analysis tool based on the amplified sequencing data. The KEGG database annotated 68 KEGG orthologs (KOs), showing significant differences between the two groups (*p* ≤ 0.05; Figure 7). There are 58 functional genes (5, 23, and 30, respectively) at the three levels (L1, L2, and L3) which were affected, with higher absolute abundances in the intercropping soil sample, especially those responsible for the metabolism, transporters, ABC transporters, general function prediction only, and DNA repair and recombination proteins (Figures 7A,C). Meanwhile, we observed the difference in increased functional genes that were counted a high percentage of metabolism categories at the L3 level, namely, amino acid metabolism, carbohydrate metabolism, nucleotide metabolism, lipid metabolism, energy metabolism, and metabolism of cofactors and vitamins (Figure 7B), whose results showed that intercropping improved the function of soil bacteria, especially the metabolic activity of bacteria.

The pathway of biosynthesis of type II polyketide backbone and melanogenesis were significantly higher in the intercropping group (Figure 7D). Polyketides are a large class of secondary metabolites with diverse chemical structures and rich biological activities, including many antibiotics, antifungal agents, cell inhibitors, anticholinergic binders, antiparasitic agents, animal growth promoters, and natural pesticides, which were widely used in clinical or other fields. The significant difference in the biosynthesis of type II polyketide skeleton between LP and CK may be closely associated with the resistance to soil-borne disease under intercropping treatment. Similarly, the remarkable difference in replication and repair genes in absolute abundance between two treatments performed the same trend (Figure 7B).

## Discussion

### Effect of Intercropping on Soil Properties

A large number of research confirmed that N can be transferred to host plants by legumes in intercropping patterns (Salgado et al., 2020). It is reported that nearly 2.0% N was transferred from *Trifolium repens* to citrus *via* common mycorrhizal networks (CMNs) with the tracer method (Fang et al., 2020). Kessel and Roskoski (1988) proved the N-transfer from *Glycine max* to *Zea mays* with the ^15^N-labeled method. Ledgard et al. (1985) found that 2.2% N was transferred directly from *T. repens* to *Lolium perenne*. During the growth period of crops, the application of N fertilizer could lead to the phenomenon of “Nitrogen repression (inhibitory effect of nitrogen application on N_2_ fixation),” namely, the reduction of nodulation and biological nitrogen fixation (BNF) in the legume (Li et al., 2009; Chai et al., 2017). However, a series of studies have shown that intercropping could improve the N fixation ability of legume crops, increase N transfer, reduce the “Nitrogen repression,” and achieve efficient N use to offset the negative effect of N application (Chu et al., 2004; Fan et al., 2006). It is deemed that graminaceous crops can stimulate the nodulation and N fixation of legume crops due to the competitive utilization of NO3^−^-N or NH4^+^-N in the rhizosphere of legume crops by gramineous plants (Corre-Hellou et al., 2005). There is an academic perspective that in the intercropping system, the soil mineral N is maintained at a low level due to the massive absorption of nitrate by gramineous plants, and the inhibition of N fixation by legumes is decreased by reducing the soil mineral N, which is considered the subtractive effect of N-repression (Xiao et al., 2003). In our study, the LP resulted in a significantly low value of AN content, considering that the N fertilizer we used was compound fertilizer (including 9.8% ${\text{NO}}_{3}^{-}$ and 12.2% ${\text{NH}}_{4}^{+}$) for only 3 periods of time in which ${\text{NO}}_{3}^{-}$N performed a larger inhibitory effect on nodulation of legumes compared to ${\text{NH}}_{4}^{+}$-N, and the plausible hypothesis may be due to the reduction of mineral N contents by “Nitrogen transfer” from Pinto peanut to litchi tree *via* CMNs; hence, the inhibition of legume N fixation could be lessened.

Soil K plays an indispensable role in increasing yield, improving fruit quality, and maintaining soil fertility (Sherif, 2018; Hamdy et al., 2019; Kheder and Abo- Elmagd, 2021; Wang W. et al., 2021). Microorganisms play a central role in the natural K cycles (Diệp and Hieu, 2013) and the K solubilizing microorganisms (KSMs), namely, *Bacillus mucilaginosus, Bacillus edaphicus, Bacillus circulans, Paenibacillus* spp., and *Acidithiobacillus ferrooxidans* (Singh et al., 2010; Rajawat et al., 2011; Basak and Biswas, 2012) are a rhizospheric microorganism which solubilizes the insoluble K to soluble forms of K for plant growth and yield (Meena et al., 2014). Researchers found that some bacterial strains could exhibit K-solubilization, promoting the absorption of K by plants (Subhashini, 2015; Kaur et al., 2021). Moreover, it is generally accepted that the major mechanism of mineral K-solubilization is the action of organic acids synthesized by rhizospheric microorganism (Aleksandrov et al., 1967; Meena et al., 2014). In our study, the absolute abundance of *Acidobacteria, Bacillus, Pseudomonas*, and *Burkholderia* was higher in LP (Figure 5), and the content of AK was significantly higher in intercropping treatment due to the influence of root system of *A. pintoi* by KSMs, which promoted the solubilization of soil insoluble K and absorption of AK by litchi.

In our previous research, we compared the soil nutrient and soil enzyme activities of the soil layer of 0–10, 10–20, and 20–30 cm in the intercropping soil of *A. pintoi* in *Areca catechu* L. Since we found that the 10–20 cm soil layer performed the most significant increase in soil nutrients and soil enzyme activities among the 3 soil layers (Yan et al., 2021), we had chosen the soil layer from 10 to 20 cm in this study. The difference of other soil properties, such as TP and AP between LP and CK, needs to be further verified considering the standard error number in Table 1 due to the deficiency of replication of samplings, which furthermore experiments should be established.

### Effect of Intercropping to Soil Enzyme Activities

Soil enzyme, a kind of biocatalyst produced in the soil, which take part in the process of transforming soil organic compounds and decomposing animal and plant residues, is one of the essential indexes of soil fertility (Li et al., 2018; Zhang et al., 2020). Among them, SC participates in the degradation of soil organic matter, acid PT accelerates the N cycle of the soil, UE participates in the transformation of N, CAT can decompose harmful substances, and PPO is involved in the aromaticity cycle (Acosta et al., 2003; Chen et al., 2018; Kuscu et al., 2018; Thapa et al., 2021). In this experiment, as the activities of SC, acid PT, UE, CAT, and PPO in litchi orchard intercropped with *A. pintoi* were significantly higher, especially in relation to the activities of SC and acid PT, we speculated that the interpenetration of peanut root system affected soil porosity and aeration (Rodriguez et al., 2020) that caused soil microbial environment changes by root exudates which enhanced soil enzyme activities. Combining with the vital role of soil K in maintaining soil fertility, we speculated that intercropping may be capable of improving soil fertility and transferring N into the soil.

### Effect of Intercropping to Soil Microbiota Diversity

Intercropping in orchard provides a novel method of weeds control, which could reduce or avoid the use of chemical herbicides and improve microbial community functional diversity (Wu et al., 2014; Sannagoudar et al., 2021; Tataridas et al., 2022). Liu et al. (2021) found that intercropping alfalfa with oat provided rich nutrients for soil microorganisms and ultimately affected the change of soil microbial dominant population and quantity due to the litter and root exudates. A higher soil microbial diversity index is more conducive to improving the stability and resistance of soil ecosystems, ensuring the normal operation of soil ecosystem function (Li et al., 2020b). The α-diversity indices, including Chao1 and ACE, reflected the richness of the soil bacterial community, and the Shannon index reflected the evenness of soil bacterial community, which was positively correlated with the diversity of the soil bacterial community (Fan et al., 2021). The above indexes were all effectively increased in this study, suggesting that the intercropping of *A. pintoi* in litchi induced a long-lasting positive impact on soil ecological environment and soil microbial diversity.

### Effect of Intercropping to Soil Microbiota Community Structure

The change in soil environment is reflected by the difference in the abundance of each group of bacteria, and increasing soil microbial populations is a key determinant in sustaining microbial functional activity (Weller et al., 2002). Some researchers have proposed that *Proteobacteria* mostly exist in the soil environment with higher nutrition (Yergeau et al., 2012). As an important microorganism in plant rhizosphere soil, *Actinobacteria* play a crucial role in promoting plant growth and controlling plant diseases (Doumbou et al., 2005). *Nitrososphaera*, a kind of beneficial microorganisms, plays an important role in ammoxidation and facilitates the N cycling in soils (Spang et al., 2012). It was reported that the abundance of *Candidatus Nitrososphaera* and *Bacillus* was much higher in the intercropping system (Gao et al., 2019). Similarly, *Bacillus* is a group of plant growth-promoting bacteria, which produces antifungal compounds and phytohormones in disease-suppressive soil (Lim and Kim, 2009). We found that the absolute abundance of *Proteobacteria, Nitrososphaera, Bacillus*, and *Actinobacteria* was significantly higher, while that of *Candidatus_Koribacter* was significantly lower in intercropping pattern. Taking the above results into consideration, the positive change in soil prosperity and the assumption that the intercropping may be capable of improving the resistance of litchi root systems to soil-borne diseases are consistent with previously reported findings (Morris et al., 2017; Zhou et al., 2019).

*Acidobacteria* is widely distributed in the soil and has a role in recovering soils as beneficial to soil nutrient cycling and plant growth (Kielak et al., 2016). The correlation between the abundance of *Acidobacteria* and soil pH was expounded completely inequable in different studies that some researchers consider that the abundance of *Acidobacteria* is positively related to soil pH, while the others hold the opposite viewpoint (Jones et al., 2009; Lauber et al., 2009; Griffiths et al., 2011). In our research, the absolute abundance of *Acidobacteria* increased significantly in the intercropping group; nonetheless, there is no obvious change in soil pH, which was consistent with the results of Liu et al. (2015). These differences may be due to the different responses of subpopulations of acid bacilli or the different acid bacilli in the same subpopulations to soil pH.

### Correlations of Soil Properties With Soil Bacterial Communities and Soil Enzyme Activities

In previous study, N is usually the most important growth factor for plants and soil microorganisms (Zhang et al., 2020). Interestedly, we found that soil AK is the vital factor leading to changes in bacterial communities in this study by the analysis of RDA combined bacterial species richness and diversity indices with chemical properties of soil at both genus and phylum levels (Figure 6). At the phylum level, AK had a significant correlation with *Actinobacteria, Proteobacteria*, and *Bacteroidetes*, while negatively related to *Acidobacteria* that *Acidobacteria* is an index of soil environment with poor nutrition (Dion, 2008). At the genus level, AK had a strongly positive impact on *Flavobac, Rhodopln, Gaiella, Nitrosos*, and *Pseudolb*. Moreover, as shown in Figure 6, the primary factor affected soil property was changed from pH (at the genus level) or AN (at the phylum level) in the monoculture groups to AK (both at genus and phylum levels) in the intercropping group. These can be interpreted as the conclusion that intercropping can increase soil K content and soil fertility by improving soil bacterial community structure, especially the absolute abundance of eutrophic bacteria. Meanwhile, there was a positive correlation between *GP4, GP5, GP6*, and AK, and a negative correlation between *GP1, GP2, GP3, GP7*, and AK, which suggested that different phyla of *Acidobacteriaceae* could regulate soil K in the soil. In addition, pH, TP, and TK were also important for regulating bacterial distribution. For example, pH and TP contributed to *GP1, GP2, GP3*, and *Bacillus*, while TK was strongly related to *Nitrosos* and *GP4*. This indicates that pH and AP had similar influences on soil microbial communities. Overall, the bacterial community appeared to be sensitive to slight variations in environmental factors.

Soil enzyme activity is closely related to soil properties, and there are studies suggesting that the changes in the availability of soil nutrients could alter the soil enzyme activities (Marschner et al., 2003). In accordance with Zhang et al. (2020), the AN was significantly negatively correlated with SC, UE, and CAT, while AK is significantly positively correlated with PPO and SC in our study, which indicated that the high level of soil enzyme may promote the synthesis and transportation of soil properties, such as AK.

### Intercropping Was Speculated to Promote the Resistance to Soil-Borne Diseases by Improving the Function of Soil Bacteria of Metabolic Activity

Root-secreted secondary metabolites are known to shape the soil microbial community, especially in the recruitment of beneficial microbes and suppression of soil-borne pathogens (Zhang et al., 2013; Yang et al., 2014), because different plant species secrete distinct root exudate profiles that stimulate different types of soil microorganisms (Hartmann et al., 2008; Zhao et al., 2017). It is reported that the *Fusarium* wilt of watermelon under the intercropping system was substantially suppressed as the growth and population of *Fusarium oxysporum* f. sp. *niveum* (FON) was reduced with the application of root exudates of wheat (Lv et al., 2018). In our study, the function of soil bacteria was improved in intercropping groups, especially the metabolic activity. We speculated that the root interaction between litchi and Pinto peanut may increase the metabolic activity level of root exudates in the soil to suppress soil pathogenic microorganisms. In addition, researchers have discovered that K-solubilizing bacteria can suppress pathogens (Lian et al., 2002), and the higher absolute abundance of KSMs, namely, *Acidobacteria, Bacillus, Pseudomonas*, and *Burkholderia* in LP is consistent with the above speculation.

## Conclusion

Our study demonstrated the difference in soil properties, soil enzyme, and soil bacterial community between monoculture pattern of litchi and over 4 years of intercropping Pinto peanut in litchi orchard. In summary, soil AK content of intercropping soil is significantly higher than that of monoculture of litchi, and soil microbial structure is significantly optimized in LP as AK is the vitally dominating environmental factor that affected bacterial community diversity with the analysis of RDA. Since intercropping has lower N contents, the dose and frequency of N application should be closely monitored in litchi orchard with a long-term intercropping of Pinto peanut. The activities of SC, acid PT, and UE were higher in LP than in CK. Intercropping showed a higher richness and diversity of soil bacteria than CK, and the structure of the soil bacterial community was obviously optimized to intercropping pattern, which is conducive to superior soil environmental conditions for litchi cultivation. By the analysis of functional predictions for soil microbiota, resistance to soil-borne disease was speculated to be highly correlated with the soil bacterial community structure in intercropping pattern. Based on the difference in AK and AN contents between LP and CK, further studies are needed in the long term of dynamic change in soil properties and microbial community.

## Data Availability Statement

The datasets presented in this study can be found in online repositories. The names of the repository/repositories and accession number(s) can be found at: https://www.ncbi.nlm.nih.gov/bioproject/PRJNA803871.

## Author Contributions

YZ, HF, and FH designed the research. YZ, CY, and SZ performed the experiment and data analysis. YZ wrote the manuscript. YZ, HF, and ZC revised and commented on the draft. All authors read and approved the final manuscript.

## Funding

This research was supported by The Major Science and Technology Projects of Hainan Province, China (ZDKJ2021006), the Major Program of The National Key Research and Development Program of China (2017YFD0202100), and The Key Research and Development Program of Hainan Province, China (ZDYF2018236).

## Conflict of Interest

The authors declare that the research was conducted in the absence of any commercial or financial relationships that could be construed as a potential conflict of interest.

## Publisher's Note

All claims expressed in this article are solely those of the authors and do not necessarily represent those of their affiliated organizations, or those of the publisher, the editors and the reviewers. Any product that may be evaluated in this article, or claim that may be made by its manufacturer, is not guaranteed or endorsed by the publisher.
