# Supplementary figures and images for: Intercropping Pinto Peanut in Litchi Orchard Effectively Improved Soil Available Potassium Content, Optimized Soil Bacterial Community Structure, and Advanced Bacterial Community Diversity
Source: Front Microbiol. 2022 May 12;13:868312. doi: 10.3389/fmicb.2022.868312 (PMC9134032; doi:10.3389/fmicb.2022.868312)

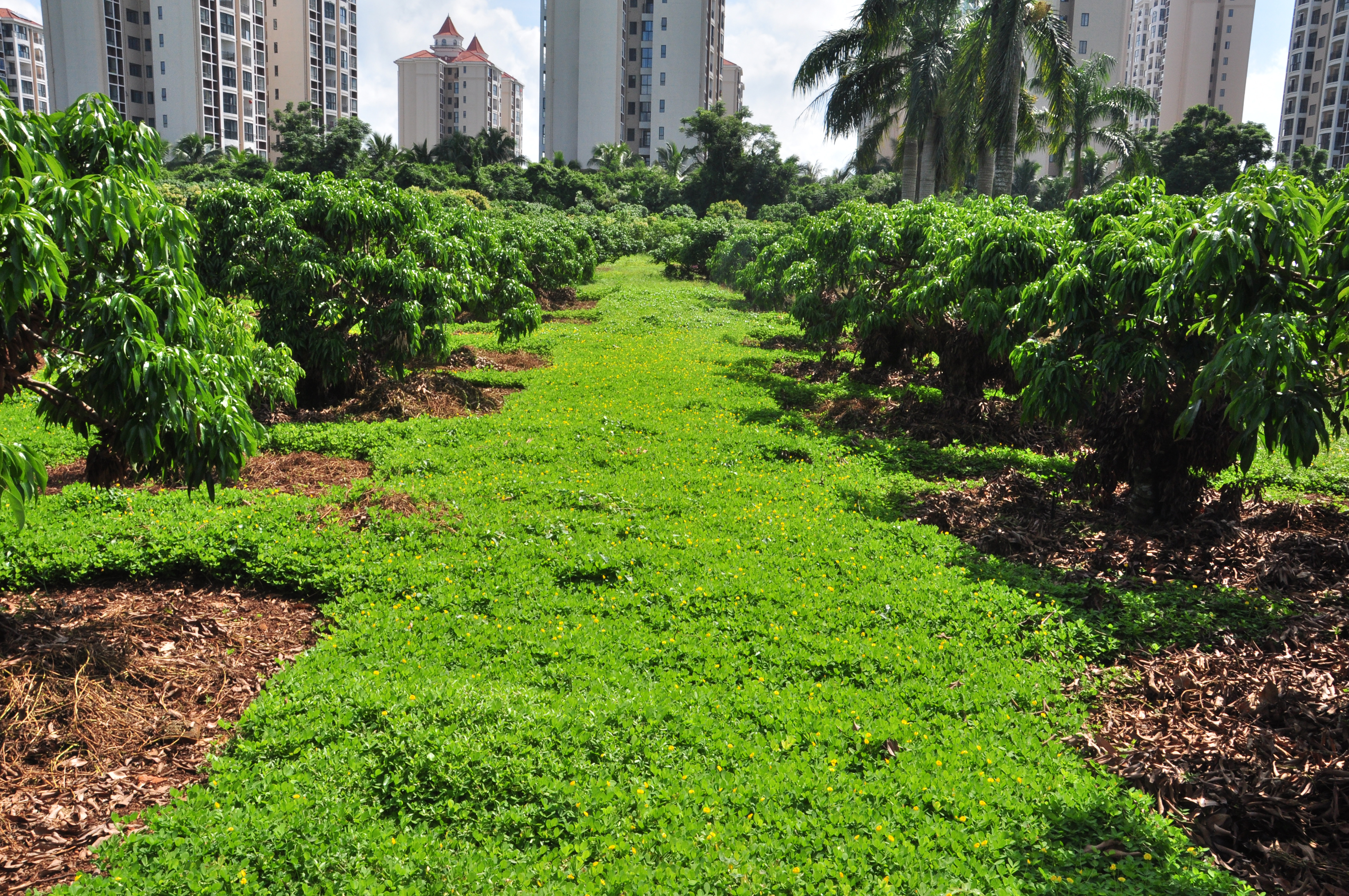

Supplement: Supplementary file 2 [file Image_1.JPG]
